# Anodically Grown Titania Nanotube Induced Cytotoxicity has Genotoxic Origins

**DOI:** 10.1038/srep41844

**Published:** 2017-02-06

**Authors:** M. Sheikh Mohamed, Aida Torabi, Maggie Paulose, D. Sakthi Kumar, Oomman K. Varghese

**Affiliations:** 1 Bio Nano Electronics Research Centre, Graduate School of Interdisciplinary New Science, Toyo University, Kawagoe, 350-8585 Japan; 2 Nanomaterials and Devices Laboratory, Department of Physics, University of Houston, Houston, Texas 77204, USA

## Abstract

Nanoarchitectures of titania (TiO_2_) have been widely investigated for a number of medical applications including implants and drug delivery. Although titania is extensively used in the food, drug and cosmetic industries, biocompatibility of nanoscale titania is still under careful scrutiny due to the conflicting reports on its interaction with cellular matter. For an accurate insight, we performed *in vitro* studies on the response of human dermal fibroblast cells toward pristine titania nanotubes fabricated by anodic oxidation. The nanotubes at low concentrations were seen to induce toxicity to the cells, whereas at higher concentrations the cell vitality remained on par with controls. Further investigations revealed an increase in the G_0_ phase cell population depicting that majority of cells were in the resting rather than active phase. Though the mitochondrial set-up did not exhibit any signs of stress, significantly enhanced reactive oxygen species production in the nuclear compartment was noted. The TiO_2_ nanotubes were believed to have gained access to the nuclear machinery and caused increased stress leading to genotoxicity. This interesting property of the nanotubes could be utilized to kill cancer cells, especially if the nanotubes are functionalized for a specific target, thus eliminating the need for any chemotherapeutic agents.

Recent advances in the synthesis and development of nanomaterials (NMs) of diverse origins, structures and properties have immensely impacted various fields ranging from consumer electronics to medical diagnosis and therapy. At such a rapid pace of NMs’ intrusion into virtually every aspect of life, the concern over their possible adverse or deleterious effects on life forms has become a major topic of discussion^1^. As the NMs differ widely with respect to their physicochemical parameters such as size, shape and elemental constituents, analysis of their toxicity involves complex challenges^2^. In addition, cell culture conditions, cell age and cell density can also influence the toxicity of the nanostructures^3^. Research so far has revealed different modes of NMs induced toxicity including the induction of oxidative stress due to reactive oxygen species (ROS) generation, inflammation, genetic damage and arresting of cell division, all culminating in cell death^4^,^5^,^6^,^7^. Different routes of exposure have also been shown to cause NM accumulation in major organs and induce toxicity^8^,^9^,^10^,^11^,^12^,^13^,^14^.

Among the various kinds of NMs currently in focus, the nanotubes (NTs) have been proven most useful for many applications due to their architecture derived unique functionalities^15^,^16^. The tubular shape, inner and outer surfaces and the nanoscale wall, all provide them distinct advantages over other nanoarchitectures. A prerequisite for their *in vivo* use is an in-depth understanding on their toxicological effects. The toxicity of nanoparticles of iron oxide, gold and cadmium chalcogenides, were investigated but the studies were limited to nearly isotropic particles^17^,^18^,^19^ (*i.e.*, with a low aspect ratio compared to fibers). Most studies on the bio-applications and toxicity have so far almost been exclusively focused on carbon based NTs^20^,^21^,^22^,^23^,^24^,^25^,^26^. Similar studies were also reported on other one dimensional materials including silver nanowires (NWs), silicon NWs and germanium-imogolite nanotubes^27^,^28^,^29^,^30^. Although there were some attempts (discussed later), the toxicity of oxide nanotubes^31^ such as titanium dioxide (TiO_2_) NTs has not been addressed in detail. We note that titanium and its alloys are currently the most widely employed materials for dental and orthopedic implants owing to their non-toxicity and biocompatibility along with commendable mechanical properties and corrosion resistance^32^,^33^.

While titania nanotubes can be fabricated by a number of physical, chemical and electrochemical methods, the self-assembled nanotube arrays grown by anodic oxidation has received incredible attention due to the ease in fabrication as well as the exceptional structural, electrical, thermal and optical properties exhibited by them^34^,^35^,^36^. These nanotubes find potential use in *in-vivo* applications also. In a number of studies on the cell response to TiO_2_ nanotubes, nanosize effects were demonstrated for a variety of cells^37^,^38^. It was reported that the surface topography of nanoscale TiO_2_ strongly affected the proliferation, migration, and differentiation of mesenchymal stem cells and hematopoietic stem cells, as well as the behavior of osteoblasts and osteoclasts^39^ which prevailed even after coating the nanotubes with an alloy^40^. It was established that the geometric factors of nanotubes greatly influenced cell vitality. We note that most of the so called ‘biocompatible materials’ including titanium and its alloys, have shown to instigate inflammatory cell recruitment, granulation tissue formation, foreign body reaction, fibrosis and other related biological events^41^,^42^. Such unwarranted events may lead to poor skin–material integration, which is one of the major causes for transcutaneous implantable devices’ rejection and undesired side effects^43^,^44^,^45^. Therefore, it is of extreme importance that nanomaterials, regardless of their physicochemical characteristics, be thoroughly investigated under *in vitro* and *in vivo* conditions prior to human and environmental exposure. Herein, we discuss the results of our comprehensive study on the anodically grown TiO_2_ nanotubes for their compatibility towards human dermal fibroblasts. We used a variety of assays ranging from cell-vitality to mitochondrial, cytoskeletal and nuclear integrity. Our investigations revealed for the first time the titania nanotube induced cyto/genotoxicity.

## Results and Discussion

### TiO_2_ Nanotubes

**Figure 1:**
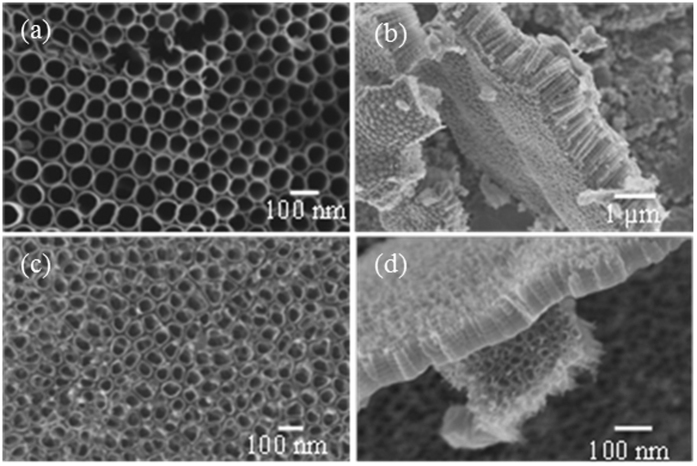
SEM images of TiO_2_ nanotube array films separated from substrates. (**a**) Top and (**b**) lateral views of the nanotubes fabricated using the EG/NH_4_F electrolyte. (**c**) and (**d**) show the images of the corresponding regions in films grown in the water/HF electrolyte.

**Figure 2:**
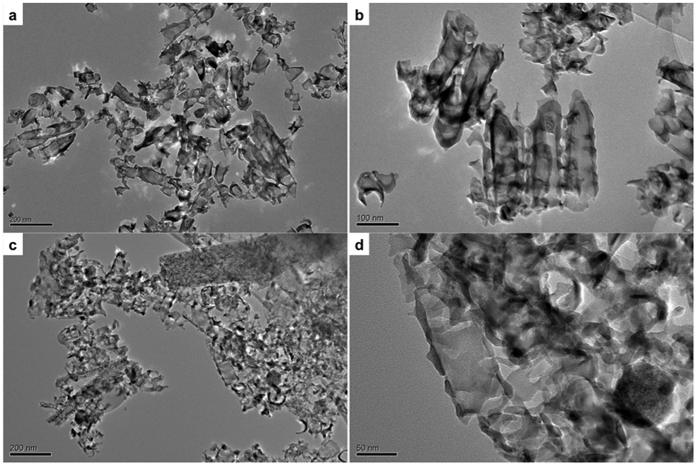
TEM micrographs of TiO_2_ nanotubes. (**a**) Low magnification and (**b**) high magnification images of ethylene glycol (organic) electrolyte fabricated TiO_2_ nanotubes. (**c**) Low magnification and (**d**) high magnification images of HF/H_2_O (aqueous) electrolyte fabricated TiO_2_ nanotubes.

As discussed in detail in Materials and Methods, we used titania nanotube arrays anodically grown in hydrofluoric acid (HF)/water and ethylene glycol (EG)/Ammonium fluoride (NH_4_F) electrolytes for the study. Fig. 1 shows the field emission scanning electron microscope (SEM; LEO 1525) images of the as-fabricated nanotube arrays. The nanotubes grown in HF/H_2_O electrolyte had an average length of 146 nm and pore diameter 35 nm and those grown in EG/NH_4_F electrolyte had these dimensions 970 nm and 70 nm respectively. These nanotube arrays, after heat treatment and detachment from the substrate, were sonicated in water to obtain dispersed individual nanotubes for further experiments. The transmission electron microscope (TEM; JEOL JEM-2100) images of these dispersed nanotubes are shown in Fig. 2. It is evident from the TEM images that nanotubes were broken during detachment and were of similar length (~150 nm) regardless of the fabrication conditions. The NTs were devoid of any metal substrate elements as NT array films were detached electrochemically from the substrates (see Materials and Methods).

The anodically grown NTs are generally amorphous upon formation and are known to crystallize at ~280 °C with anatase phase formed in the NT walls^46^,^47^. The selected area diffraction pattern and the lattice image showing polycrystalline anatase phase present in the wall of an NT prepared using EG/NH_4_F electrolyte and heat treated at 530 °C are given in Supporting Information Fig. S1. The HF and EG based NTs used in the present study were crystallized by heat treating at 500 and 450 °C respectively.

### Cytotoxicity Analyses

**Figure 3:**
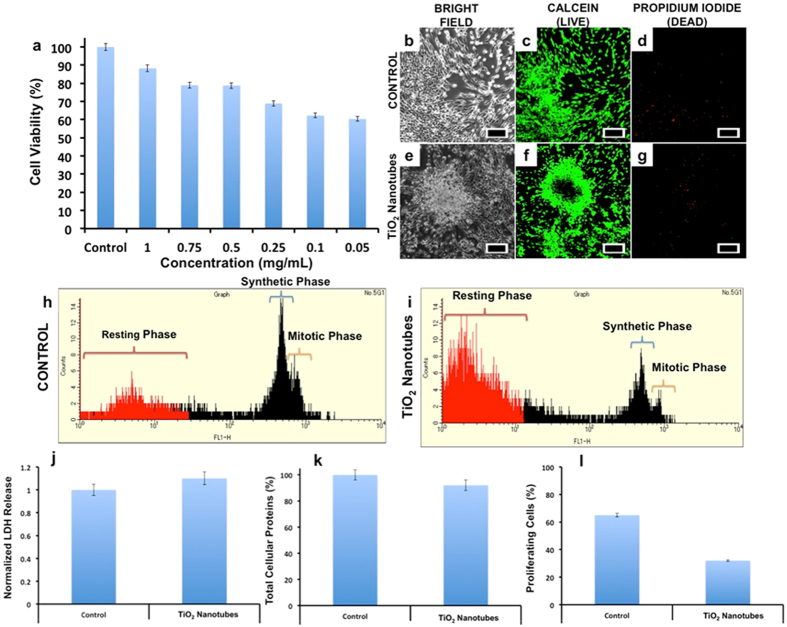
Cellular toxicity analysis for the effects of TiO_2_ nanotubes on human dermal fibroblasts. The alamar blue assisted assay revealed a dose independent viability chart, with viability decreasing with decrease in concentration (**a**). Though an overall maximum viability remained at approximately 60%. (Columns, means of three independent experiments; bars ± SD). The live dead analysis (**b–g**) revealed a generally live cell population in the NTs treated group (**e–g**) which could be correlated to the residual esterase even in the cells undergoing apoptosis (**f**) (scale bar represents 500 μm). In the cell cycle analysis a predominant synthetic and mitotic phase was observed with the control cells (**h**), however in the NTs group a prominent resting phase was observed (**i**). The LDH release was found to be insignificant in treatment group when compared to control (**j**); The TCP expression were found to be unaffected (**k**) whereas the number of proliferating cells in the treatment group dropped by half when compared to control (**l**).

Nanotubes generally share morphological similarities with asbestos fibrils, which are linked to progressive fibrotic ailments^48^. On this basis we explored the acute cytotoxicity of NTs on human dermal fibroblast cells (HDAF). The effect of NTs on the proliferation and metabolic status of the cells was first investigated by alamar blue assay. The NTs fabricated by both aqueous and organic electrolytes were slightly toxic at lower concentrations (0.05, 0.1 and 0.25 mg/mL) when compared to the higher concentrations (0.5–1.0 mg/mL) after the incubation period of 72 h. In the case of samples fabricated using EG electrolyte, the viability reduced to 60.37, 62.26 and 68.86% for 0.05, 0.1 and 0.25 mg/mL NTs treated set, respectively (Fig. 3a). Higher concentrations i.e., 0.5, 0.75 and 1.0 mg/mL, showed 78.77, 78.94 and 88.22% viabilities, respectively. Although the nanotubes fabricated by HF/H_2_O electrolyte were significantly shorter (by a factor of ~6) originally, similar results were obtained from these nanotubes also (see Supporting Information Fig. S2). Nonetheless, as mentioned earlier, the dimensions of nanotubes prepared in both the electrolytes became similar after sonication. It is reasonable to believe that the similarity in the toxic nature of both types of nanotubes arises from their comparable dimensions. The study also confirmed that the nanotube pedigree had no apparent role in determining the toxicity. For these reasons, we used the samples prepared using EG/NH_4_F electrolyte for all subsequent studies.

The broken NTs were found to be aggregated and difficult to disperse by sonication. Post sonication, uneven polydisperse size distribution in solutions was evident from Fig. 2a. This observation was similar to earlier studies on carbon nanotubes, where the length and morphology of the carbon nanotubes were distorted after sonication^49^,^50^. The extent of aggregation increased when NTs were added to the cell culture media which could likely have prevented cellular cytosol from being exposed to individual NTs at higher concentrations. It is worth mentioning that the NTs were bare and not surface functionalized with biocompatible/surfactant coating, which not only enhances the compatibility of a material but also renders significant and uniform dispersion of the nanomaterial under investigation. Due to the bare nature of NTs, exposure to cell culture medium (containing serum and various proteins) induces aggregation and formation of large clusters that cannot be efficiently endocytosed by cells and therefore may not have shown toxicity. Whereas at lower concentrations, the clusters are expected to be within the endocytosis limit, leading to relatively higher uptake by cells facilitating interaction with intracellular components. Though the dosing metric is considered to be a common way to assess viability, the method is complicated because very little is known about how the aggregation behavior of the NTs affects the dosage/concentration-based toxicity. TiO_2_ nanoparticles and nanotubes tend to aggregate easily in solution when not surface modified. Such aggregation behavior was reported in a few uptake and localization studies^51^,^52^,^53^,^54^. Though several studies were reported on toxicity analysis of TiO_2_ NTs, not much was discussed about the dosage-based aggregation of the NTs. In most of the reported studies, contradictory results were presented with some finding NTs as highly biocompatible, while some others observing toxicity in them^55^. TiO_2_ nanomaterials, especially the architectures with high aspect ratio such as NTs, tend to aggregate in solution. This characteristic made preparation of the dosing solutions in this study difficult because the conventional mechanical dispersion methods such as sonication provided only polydispersed solutions, which contained a mixture of individual nanotubes, nanotube aggregates, and particle debris that might have contributed to the interesting results presented here.

We point out that the nanotubes synthesized with aqueous as well as organic electrolytes, showed a similar toxicity profile, i.e., lower viability at lower concentration and higher viabilities at higher concentrations. The TEM images clearly display the fractured forms of nanotubes (Fig. 2). Though both nanotube groups differed in their structural dimensions immediately after synthesis, their morphological features became similar on sonication. We did not see any distinct difference in the cytotoxicity profiles of amorphous and polycrystalline nanotubes for a given nanotube concentration. Furthermore, as the metal substrate elements were completely removed from the nanotubes during detachment, their role in the recorded toxicity could be completely ignored. Therefore, the observed interesting cytotoxicity profile could be correlatively attributed to the size and dimensions of the test materials. The aggregation behavior was visible through microscope as well as with naked eye. The large clusters were seen to be floating in the media or attached to the cell/substratum. Therefore, it was concluded that the larger aggregates remained ineffective while the smaller aggregates or individual broken NTs specifically gained access to the cellular interior and exerted the effects observed thereof.

To assess the possible reasons for the observed toxicity of NTs based on alamar blue analysis, 0.05 mg/mL of the nanomaterial was used for all further experiments. Fluorometric live/dead cell viability and fluorometric cell cycle analysis were assayed. Using the cell viability assay that stains individual cells and measures the number of live versus dead population, it could be observed that most cells exhibited esterase staining, depicting viability (Fig. 3c,f). With alamar blue based cytotoxicity analysis, we found that nearly 40% of cell population was dead when exposed to 0.05 mg/mL of NTs after 72 h. However, with live/dead staining, almost all the cells exhibited bright green fluorescence, which we believe is from the residual esterase in the cells. It could be seen that the cells were aggregated with reduced cellular volume depictive of stress conditions (Fig. 3e), which eventually culminates in cell death. Very few cells in treatment group exhibited propidium iodide (PI) staining (Fig. 3g) which confirms that the major mode of cell-death is via the apoptotic pathway rather than necrotic. To understand whether NTs could disrupt the cell cycle progression, we analyzed the cell cycle phases of the control and treatment groups. Control group cells (Fig. 3h) were predominantly seen in synthetic and mitotic phase (S and M phase), whereas very few cells were seen in resting phase (sub G_0_ phase). However, with test group (Fig. 3i), we observed a clear decline of cell population in S and M phase and a relative increase in sub G_0_ phase. The cells in sub G_0_ phase are indicative of cells which are no longer active part of cell-cycle.

Lactate dehydrogenase (LDH) is an important cytosolic enzyme and its release is considered to be a vital tool to understand the extent of plasma membrane damage when cells are exposed to toxic substances^56^. Assessing the cell membrane integrity is one of the most common ways to measure cell viability and cytotoxic effects. When tested, the NTs exposed cells exhibited a slight increase in the LDH release as compared to the control groups (Fig. 3j); however, the release was insignificant. If correlated with live/dead analysis, the esterase activity remained active and the cells exhibited bright green fluorescence indicative of their viable status. If the plasma membrane integrity were compromised, the esterases would have leaked out, leading to meager viable fluorescence. Furthermore, in this case the PI would have gained nuclear access exhibiting necrotic signals; the results obtained with live/dead assay clearly supports the retention of plasma membrane integrity that is observed with the LDH assay.

Total cellular proteins (TCP) constitute about 35% of cell volume and their expression could be affected when cells exhibit distress signals^57^. When the TCP were quantified (Fig. 3k), the amount of proteins expressed by treatment group remained very much comparable to the control group. Though a slight decrease in protein concentrations is accounted, we believe the values are insignificant and the decrease can be attributed to treated cells that are rich in resting phase rather than in synthetic phase as seen in controls.

Cell proliferation analysis kinetics is considered as an important parameter that can critically explain about the cell’s metabolic behavior at a given time^58^. EDU based cell proliferation assay is considered to be easy and reliable when compared to radioactive thymidine or BrDU based analysis. EDU binds to the newly actively synthesizing DNA by click chemistry. Nearly 65% of the controls exhibited EDU staining whereas only 30% of the treated cells were positive to EDU (Fig. 3l). According to the cell cycle analyses, we believe as most of the NTs treated cells were in the resting phase with less cells in S phase, the cell proliferation ability of this group was diminished.

### Cytoskeletal Organization

**Figure 4:**
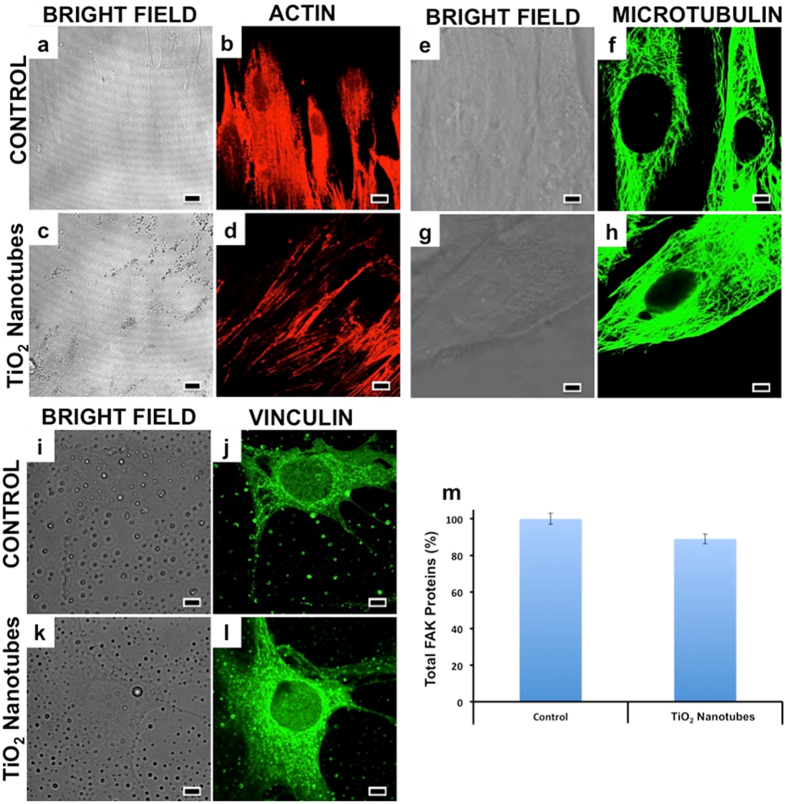
Cytoskeletal integrity analysis of the control and NTs treated group were performed with emphasis on actin, micotubulins and vinculin. With respect to the actin filaments, a slight reduction in the network of the fibres was observed with the NT group (**d**) when compared with the control (**b**). In the case of microtubulins the control (**f**) and test groups (**h**) exhibited comparable tubular network. The vinculin expression was found to be undisturbed in the treatment group (**l**) when compared to control (**j**). (Scale bars represent 10 μm). Total FAK proteins were quantified with ELISA and their expression in control and treatment groups remained comparable (**m**). Overall, the cytoskeletal components, which form the basic backbone of a cell and are responsible for their structural integrity and cell-cell/cell-matrix interactions, remained unaffected by the TiO_2_ NTs, with the exception of actin where a slight rearrangement of the fibers was observed.

Cytoskeletal disorganization could also lead to serious disorientation in organelle and protein transport within the cell’s cytosol and could fatally affect the cell’s survival^59^. A major component of cellular skeleton is actin. The actins form rigid but flexible scaffolding, facilitating the mechanical stability and mobility of cells^60^. Typically, cells are flat adherent to the substratum with strong peripheral F-actin staining along the cell edge, indicative of cell cortical actin fibers. The microtubules form a dense network that is uniformly distributed around the nucleus. Microtubules are mainly responsible for mobility and intracellular transport and their proper functioning is essential for the optimal metabolic output of a cell^61^. Compared to control cells which displayed an elaborate network of finely arranged actin fibers (Fig. 4b), cells treated with NTs exhibited F-actin thinner and less organized within the main body (Fig. 4d), however, the microtubule structures in the central and peripheral domain remained comparable with control cells (Fig. 4f,h). This result suggested a slight disorganization of the actin cytoskeleton when exposed to NTs, which could be correlated to the observations of cellular aggregation previously mentioned. This disorganization might cause a series of signal pathway disruptions, though concrete conclusions could be made only after a detailed study of downstream processes.

Vinculin, a major membrane-cytoskeletal protein, serves as focal adhesion plaque that is involved in linking integrins to the actins and is associated with cell-cell and cell-matrix junctions^62^. Vinculin disruption can cause a loss in balance within cytoskeletal organization and can lead to contact inhibition between cells and the extra cellular matrix. When investigated, the vinculin immunomapping remained same for the control and treated group (Fig. 4j,l) depicting more or less proper cytoskeletal organization.

We investigated the expression of total focal adhesion kinase (FAK) proteins that work primarily as signal-mediating molecules that direct actin based signaling pathway^63^. FAK disruption is known to affect cellular homeostasis. When quantified, the FAK levels of the treatment group were slightly decreased as compared to the control group (Fig. 4m). This approximately 8% decrease can be attributed to the actin disorganization that was previously observed. Though a slight modification in actin and FAK levels were noted, the cytoskeletal integrity remained comparable to the control groups. Earlier, single wall carbon nanotubes were shown to cause disorientation and bundling in actins *in vitro* leading to chronic changes in cell behavior without any observed acute toxicity^64^. We believe that the observed slight actin re-modeling arose from the shape-effect of the NTs rather than their intrinsic nature bound toxicity

### Mitochondrial Health

**Figure 5:**
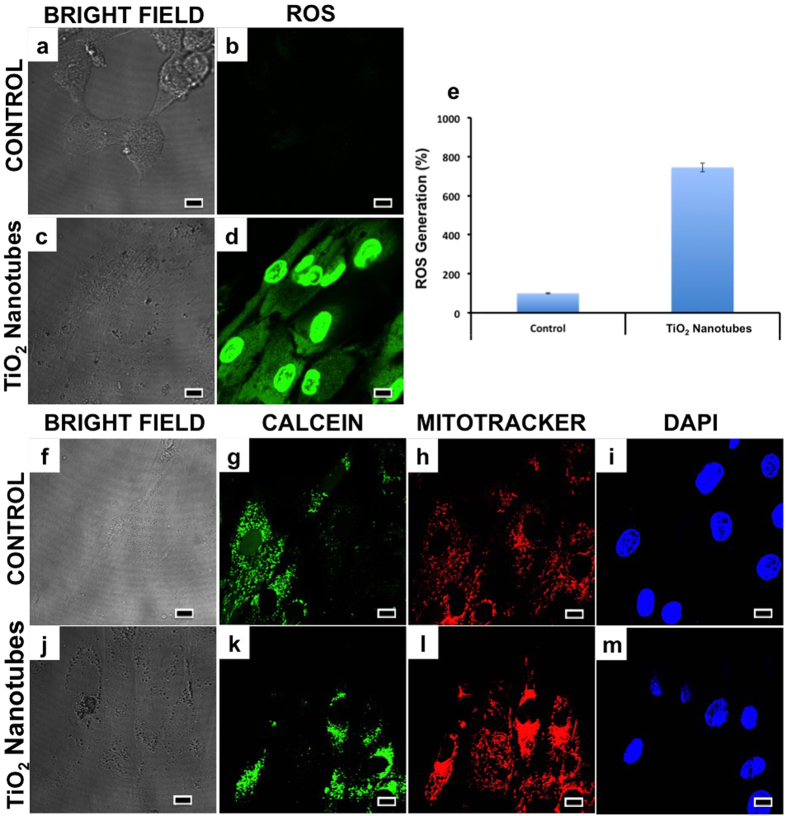
Mitochondrial integrity and condition were assayed with the ROS generation and transition pore formation tests. It was clearly evident that the NTs induced ROS production, especially nuclear ROS (**d**). The nuclear ROS in the treatment group was found to be increased by 7 fold than the control (**e**). The mitochondrial pore induction investigation revealed the absence of any distorted signals from the mitochondrial region (**l**) with discrete calcein signaling (**k**), indicative of a properly functioning mitochondrial system. (Scale bars represent 10 μm). It is evident from this assay that though the mitochondrial apparatus seems to be healthy and functioning properly, nuclear ROS is significantly enhanced on TiO_2_ NTs exposure, which may be the precursor for numerous distortions in nuclear functioning.

Earlier reports have emphasized the role played by oxidative stress in nanoparticle toxicity that causes serious oxidative damage to proteins and deoxyribonucleic acid (DNA)^1^. To establish the role of oxidative stress as a decisive factor in NT induced toxicity, ROS tracer based studies were performed. Previous studies proved a direct link between the ROS production in cells and tissues upon exposure to titania based nanostructures^65^. Untreated cells were used as standards to calculate the extent of ROS production by measuring the percentage of cells with increased fluorescence (directly corresponding to the ROS production) intensity. The analysis showed slight superoxide production in cellular cytosol, treated with 0.05 mg/mL of NTs and highly elevated levels of ROS in the nuclear compartment (Fig. 5d). As one could visualize, a dramatic increase (by 8-fold) of ROS generation could be seen in the cells that were exposed to the NTs (Fig. 5e).

Further, we investigated the mitochondrial health, as the main cause of mitochondrial dysfunction arises from ROS production and subsequent oxidative stress. Increased oxidative stress can lead to transitional pore formation in mitochondrial membrane resulting in distorted mitochondrial geometry and function^66^. The discrete calcein and mitochondrial staining in the treatment group (Fig. 5g,h) revealed a normal and functioning mitochondrial apparatus. The ROS produced due to NT induced oxidative stress was likely counteracted by anti-oxidant systems/or acted upon by other organelles (especially nucleus as observed from the image), thereby nullifying their deleterious effect on mitochondria (Fig. 5k,l).

### Genotoxicity

**Figure 6:**
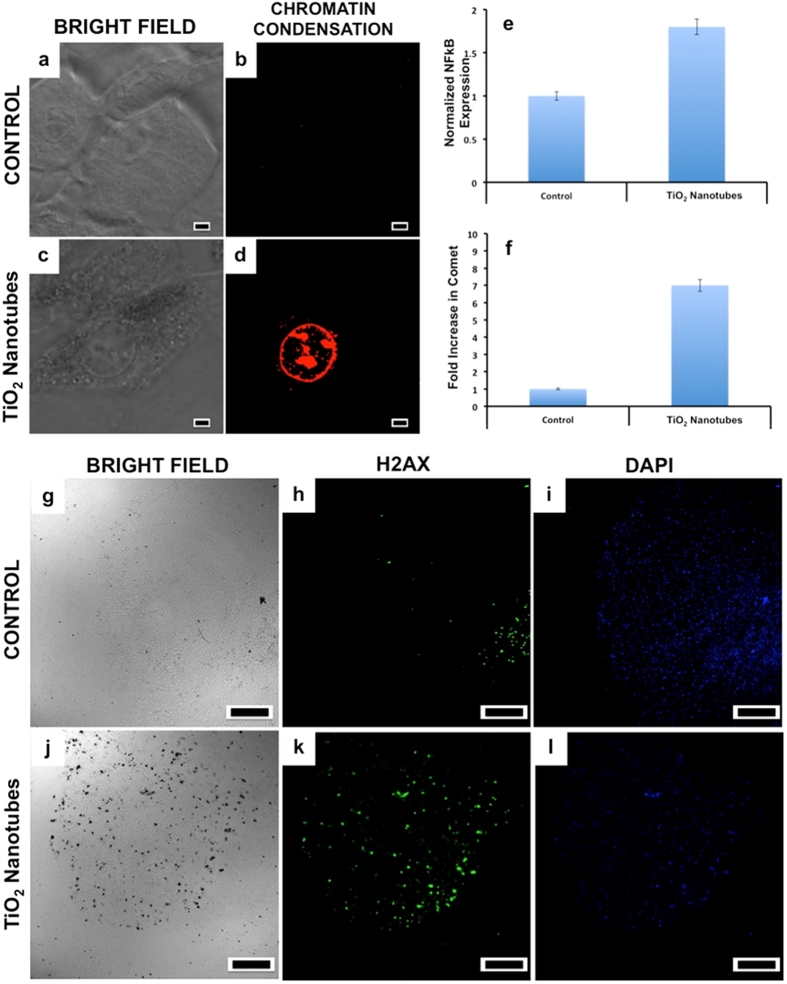
Chromatin condensation and DNA break studies were carried out to assess the condition of the nuclear set up of the control and NT treated cells. Prominent chromatin condensation and plasma membrane permeability (**c,d**) was recorded with the NTs treated group. (Scale bars represent 10 μm). The NF-κB expression was elevated in the treatment group when compared to control (**e**). Comet analysis confirmed significant increase in number of comet tails in treatment group (**f**). Consistent DNA breaks could also be observed with the H2AX test in the NTs group (**k**) when compared to the controls (**h**). (Scale bars represent 500 μm). This nuclear condensation and DNA alteration study is correlative of the ROS induction previously observed.

Genotoxicity studies that measure DNA damage such as mutations, chromosomal damage and single/double strand breaks are important part of cancer research and risk assessment of potential carcinogens. The oxidative stress, induction of high levels of ROS in nucleus upon NT exposure can cause spontaneous damage to DNA thus arresting the cell cycle progression. It was reported by Wang *et al*. that TiO_2_ NTs could gain access into nuclear region effectively^67^. To analyze whether NTs could induce genotoxicity, we assessed the chromosomal condensation and double strand DNA breaks. When compared to the normal nuclear content (Fig. 6b), cells treated with NTs for 72 h displayed nuclear chromatin condensation and compromised plasma membrane permeability (Fig. 6d) suggesting cell death via an apoptotic mode. To analyze the secondary effects of ROS, immediate mediator of ROS-induced cell death mediated signaling, NF-κB activation was profiled. As expected, nearly twice the increase in NF-κB levels were noted when compared to controls (Fig. 6e). NF-κB is considered as primary transcription factor of apoptosis that are expressed when cells are in oxidative distress. Their cytosolic expression increases immediately after an oxidative stress, and is transported to nucleus to induce apoptotic transcription leading to programmed cell death^68^. A clear correlation between the ROS levels and NF-κB expression could be made, which confirmed that the nuclear oxidative stress was the reason for observed genotoxic behavior.

To analyze whether chromosomal distortion occurred in the treatment groups, the cells were subjected to comet assay. A distorted chromosomal content, leads to tail-comet whereas the healthy chromosomal content appears spherical when subjected to comet electrophoresis. As expected, cells exposed to NTs exhibited nearly 7-fold increase in comet tail formation (Fig. 6f) again affirming the nuclear degeneration behavior of NTs. TiO_2_ nanoparticles might damage DNA directly or indirectly via oxidative stress and/or inflammatory responses. Recent studies showed a direct chemical interaction between TiO_2_ nanoparticles and DNA damage^69^,^70^. However, other studies showed that TiO_2_ nanoparticles could cause DNA damage through inflammation^71^,^72^ and/or ROS generation^73^,^74^. We performed H2AX staining, that is indicative of DNA breaks. The treatment group cells exhibited numerous DNA breaks in the nuclear compartment that are indicative of spontaneous DNA damage due to NT exposure (Fig. 6k). It was evident that oxidative stress could be sensed by the nucleus and that ROS formation was directly generated by the nucleus. Increased ROS in the nuclear region due to NT exposure attacked the nuclear components leading to genotoxicity by inducing nuclear condensation and chromosomal distortion.

## Conclusion

The results from our study indicated induction of ROS by NTs that in turn set off DNA damage and chromosomal aberrations, which are believed to be the primary factors resulting in cell cycle arrest. Cells resting at sub G_0_ interface showed no significant death, indicating the involvement of an active DNA repair pathway. Cells with a robust repair mechanism in place could re-enter the cell cycle, while those with extensive irreparable damage could not repair the DNA effectively, consequently undergoing apoptosis. The NTs displayed the potential to cause toxicity to the cells as analyzed by the cyto- and genotoxicity parameters. Keeping in mind that the NTs were not surface modified with cyto-, biocompatible moieties as polyethylene glycol etc., it is imperative that the biological applications employing NTs should be given special attention and further studies must be conducted in this field to achieve deeper understanding of NT toxicity. As mostly all the nanomaterials currently being examined for various applications including nanomedicine, pose toxicity *in vitro*/*in vivo*, and surface functionalization is mandatory, we believe that the TiO_2_ NTs utilized in the present study could hold better performance once a biocompatible coating is rendered to them. Also, as the cause of cell death is proven to be ROS instigated genotoxicity, these TiO_2_ nanotubes, after proper surface functionalization and with the assistance of cell-specific markers (antibodies/peptides) could be utilized as specific-cell killing tools, as in the case of anti-cancer therapies. For example, our current interest includes the utilization of such a properly functionalized and targeted TiO_2_ nanotube to specifically induce ROS and genotoxic effects in cancer cells and kill them as such, negating the need for any chemo agent or external stimuli.

## Materials and Methods

### Titania nanotube synthesis

The titania nanotube array samples were fabricated by anodic oxidation of titanium (Ti) foils (0.25 mm thick, 99.7% pure, Sigma Aldrich) in a two-electrode cell consisting of a titanium foil anode and a platinum foil cathode immersed in an aqueous or organic electrolyte. The aqueous electrolyte consisted of 0.5 vol% HF in deionized (DI) water. The anodization of Ti foil at 10 V for 1 h in this electrolyte yielded nanotubes having morphology shown in Fig. 1a,b. The organic electrolyte was prepared by mixing EG with 0.7 vol% ammonium fluoride (NH_4_F) and 50 vol% DI water. The nanotubes (Fig. 1c,d) were grown by anodizing Ti foil at 15 V for 1 h. The titania nanotube array samples prepared in these aqueous and organic electrolytes were then crystallized by heat treating respectively at 500 °C and 450 °C for 30 minutes in oxygen environment. The heat treated samples were anodized again in the respective electrolytes and voltages to detach the crystallized nanotubes from the metal substrate.

### Cell culture details

Human Dermal Adult Fibroblasts (HDAF) were obtained from ATCC. The cells were maintained in T25 flasks using CSC complete medium kit (Cell Systems), and incubated in a 5% CO_2_ incubator at 37 °C. The cells were sub-cultured every three days. Approximately 5000 cells were seeded into each well of 96 well plates. After attaining visual confluency, NTs were added at different concentrations (0.05 mg/mL- 1 mg/mL). Control group was devoid of NTs. After 72 h of incubation with NTs, cells were washed with PBS and 0.2 ml of respective medium was added. Cell viability was assayed with alamar blue in a 96 well plate reader (Multidetection microplate scanner, Dainippon Sumitomo Pharma). For other microplate assays, NTs at a concentration of 0.05 mg/mL were added to the cells and incubated for 72 h. Post 72 h, the cells were quantified for lactate dehydrogenase (LDH) release (Pierce LDH cytotoxicity kit), total cellular protein contents (Pierce BCA protein assay kit), total focal adhesion kinase (FAK) expression (FAK ELISA kit, Thermo Fisher), reactive oxygen species (ROS) generation (DCFH-DA Assay, Sigma) and nuclear factor-κB (NF-κB) expression (NF-κB transcription factor assay kit, Thermo Fisher) according to the respective manufacturer’s instructions.

For flow cytometry (JSAN cell sorter, Bay Bioscience) based cell cycle analysis, approximately 200,000 cells were seeded in 6 well plates and cultured for 24 h. NTs (0.05 mg/mL) were added to the cultures and incubated for 72 h. Post incubation, cells were washed in PBS and flow cytometry performed according to manufacturer’s instructions (Click-iT EDU Alexa fluor 488 flow cytometry assay kit, Life technologies).

For all confocal (CLSM, Olympus IX 81 under DU897 mode; for blue, green and red excitations, wavelength 405, 488 and 561 were used respectively) experiments, approximately 50,000 cells were cultured in 35 mm glass base dishes (Iwaki) for 24 h. The cells were incubated with the NTs (0.05 mg/mL) for 72 h. Live/dead (Live/dead cell double staining kit, Sigma-Aldrich), EDU cell proliferation (Click-iT® Plus EdU, Life Technologies), Actin (Phalloidin rhodamine, Life technologies), Microtubulin (Tubulin tracker, Life technologies), ROS (Cell-ROX, Life technologies), Mitochondrial pore induction (Mitochondrial membrane transition pore assay, Life technologies), Chromatin condensation (Life technologies), DNA break (HCS DNA damage kit, Life technologies) assays were performed as per the respective manufacturer’s instructions.

For comet assay, cells were grown until 80% confluency in T25 flasks. Treatment groups were exposed to 0.05 mg/mL of NTs whereas controls were devoid of any treatment. Post 72 h exposure, cells were collected and processed for comet assay as per manufacturer’s instructions (Alkaline Comet, Trevigen). The comet formations were counted manually and were graphed.

For all cell culture experiments, the cells were washed thoroughly with PBS after incubation with the NTs for the specified time period to remove any unbound or free floating NTs, prior to observations.

### Statistical analysis

All quantitative experiments were conducted with at least three independent experiments, each with triplicates. Statistical evaluation was performed using Origin Pro Software.

## Additional Information

**How to cite this article**: Mohamed, M. S. *et al*. Anodically Grown Titania Nanotube Induced Cytotoxicity has Genotoxic Origins. *Sci. Rep.* **7**, 41844; doi: 10.1038/srep41844 (2017).

**Publisher's note:** Springer Nature remains neutral with regard to jurisdictional claims in published maps and institutional affiliations.

## Supplementary Material

Supplementary Information
