# Supplementary material for: Anodically Grown Titania Nanotube Induced Cytotoxicity has Genotoxic Origins
Source: Sci Rep. 2017 Feb 6;7:41844. doi: 10.1038/srep41844 (PMC5292953; doi:10.1038/srep41844)
Supplement: Supplementary Information [file srep41844-s1.pdf]

Supporting Information

Anodically Grown Titania Nanotube Induced Cytotoxicity has Genotoxic Origins

M. Sheikh Mohamed[1], Aida Torabi[2], Maggie Paulose[2], D. Sakthi Kumar[*1], Oomman K. Varghese[2*]

[1] *Bio Nano Electronics Research Centre, Graduate School of Interdisciplinary New Science, Toyo University, Kawagoe, Japan- 350-8585*

[2] *Nanomaterials and Devices Laboratory, Department of Physics, University of Houston, Houston, Texas 77204, USA.*

*sakthi@toyo.jp; (+81)-(0)492-39-1636/1375/1640 (phone); (+81)-(0)366-77-1140 (fax); okvarghese@uh.edu; (1) 713 743-3808 (phone); (1) 713 743-3589 (fax).

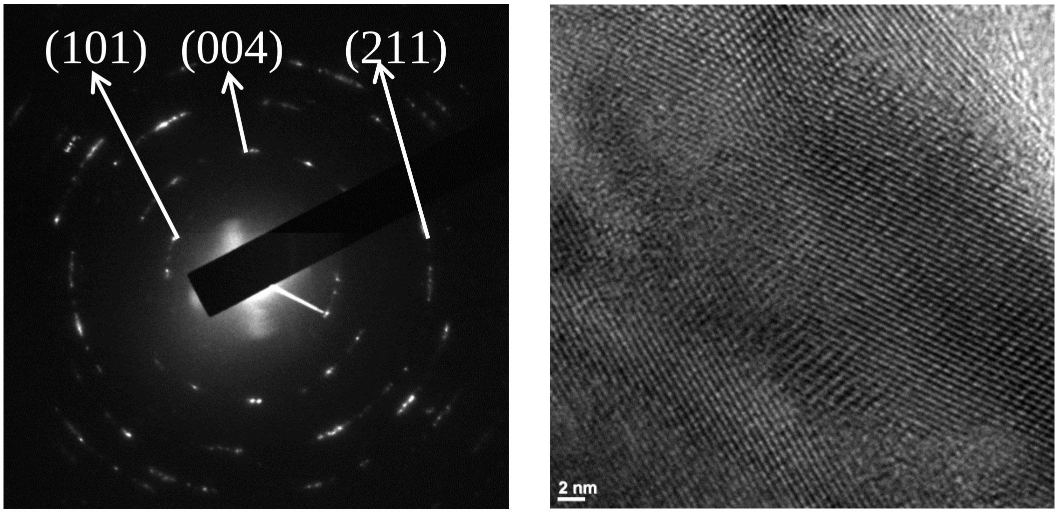

Figure S1. (Left) Selected area diffraction pattern and (**Right**) high resolution TEM (JEOL 2010) image recorded from the wall of a titania nanotube prepared using NH_4F/EG electrolyte and annealed at 530 °C for 3 h in oxygen atmosphere. Polycrystalline anatase phase is evident.

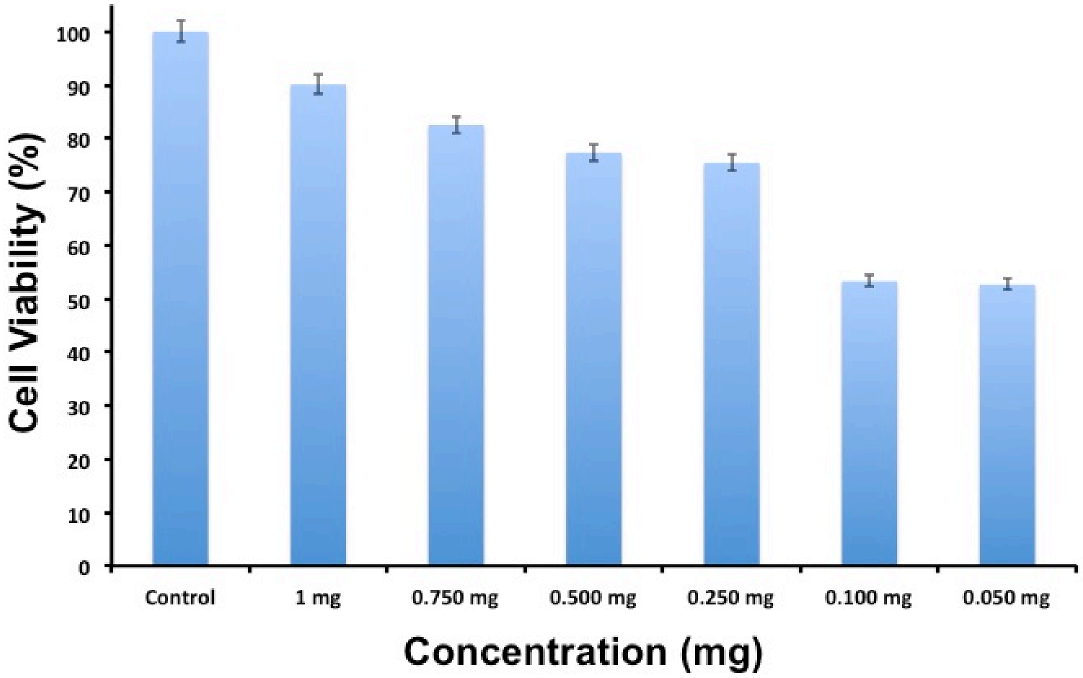

Figure S2. Cellular toxicity analysis of HF/H_2O TiO_2 nanotubes on human dermal fibroblasts. The alamar blue assisted assay revealed a dose independent toxicity, with viability decreasing with decrease in concentration as observed with the organic electrolyte fabricated NTs.
